# The prognostic impact of the immune signature in head and neck squamous cell carcinoma

**DOI:** 10.3389/fimmu.2022.1001161

**Published:** 2022-10-04

**Authors:** Hasan Baysal, Vasiliki Siozopoulou, Hannah Zaryouh, Christophe Hermans, Ho Wa Lau, Hilde Lambrechts, Erik Fransen, Ines De Pauw, Julie Jacobs, Marc Peeters, Patrick Pauwels, Jan Baptist Vermorken, Evelien Smits, Filip Lardon, Jorrit De Waele, An Wouters

**Affiliations:** ^1^ Center for Oncological Research (CORE), Integrated Personalized and Precision Oncology Network (IPPON), University of Antwerp, Antwerp, Belgium; ^2^ Department of Pathology, Antwerp University Hospital, Antwerp, Belgium; ^3^ StatUa, University of Antwerp, Antwerp, Belgium; ^4^ Department of Oncology, Antwerp University Hospital, Antwerp, Belgium; ^5^ Center for Cell Therapy and Regenerative Medicine, Antwerp University Hospital, Antwerp, Belgium

**Keywords:** squamous cell carcinoma of the head and neck (HNSCC), the Cancer Genome Atlas (TCGA), immune composition, immune checkpoint ligands, prognostic markers, clinicopathological characteristics

## Abstract

Head and neck squamous cell carcinoma (HNSCC) is a heterogeneous group of tumors that retain their poor prognosis despite recent advances in their standard of care. As the involvement of the immune system against HNSCC development is well-recognized, characterization of the immune signature and the complex interplay between HNSCC and the immune system could lead to the identification of novel therapeutic targets that are required now more than ever. In this study, we investigated RNA sequencing data of 530 HNSCC patients from The Cancer Genome Atlas (TCGA) for which the immune composition (CIBERSORT) was defined by the relative fractions of 10 immune-cell types and expression data of 45 immune checkpoint ligands were quantified. This initial investigation was followed by immunohistochemical (IHC) staining for a curated selection of immune cell types and checkpoint ligands markers in tissue samples of 50 advanced stage HNSCC patients. The outcome of both analyses was correlated with clinicopathological parameters and patient overall survival. Our results indicated that HNSCC tumors are in close contact with both cytotoxic and immunosuppressive immune cells. TCGA data showed prognostic relevance of dendritic cells, M2 macrophages and neutrophils, while IHC analysis associated T cells and natural killer cells with better/worse prognostic outcome. HNSCC tumors in our TCGA cohort showed differential RNA over- and underexpression of 28 immune inhibitory and activating checkpoint ligands compared to healthy tissue. Of these, CD73, CD276 and CD155 gene expression were negative prognostic factors, while CD40L, CEACAM1 and Gal-9 expression were associated with significantly better outcomes. Our IHC analyses confirmed the relevance of CD155 and CD276 protein expression, and in addition PD-L1 expression, as independent negative prognostic factors, while HLA-E overexpression was associated with better outcomes. Lastly, the co-presence of both (i) CD155 positive cells with intratumoral NK cells; and (ii) PD-L1 expression with regulatory T cell infiltration may hold prognostic value for these cohorts. Based on our data, we propose that CD155 and CD276 are promising novel targets for HNSCC, possibly in combination with the current standard of care or novel immunotherapies to come.

## Introduction

Head and neck squamous cell carcinoma (HNSCC) is the fifth most common cancer type, with a current 800,000 new cases/year worldwide that is estimated to reach 1 million annual cases by 2030 (1, 2). Despite advances made in conventional treatments, the 5-year survival rate of HNSCC has remained stagnant. Meanwhile, mortality rates have increased to nearly 500,000 individuals in 2018 (2). This lack of improvement is mostly attributed to the limited response rates and treatment-related toxicities observed with current treatments (3). Therefore, innovative therapeutic strategies are necessary to improve survival and limit the unwanted toxicities of conventional treatments.

Targeted therapies have seen an overwhelming interest in the last decade for their ability to inhibit oncogenic “driver” pathways. However, issues such as tumor heterogeneity and multiple resistance mechanisms following single pathway inhibition have limited the observation of durable responses (4, 5).. Interestingly, similar to some chemotherapies, alterations of some oncogenic signaling pathways are known to affect the immunogenicity, immune recognition, and subsequent elimination of cancer cells by the immune system (6, 7). In response to this antigenic nature, tumor-driven mechanisms of immune evasion circumvent or suppress immune-mediated targeting and killing. Therefore, improving the understanding of the tumor immune microenvironment (TIME) to reinvigorate the immune system through immunotherapies and more specifically immune checkpoint inhibitors may achieve long-lasting antitumor immune responses (8–10). For recurrent and metastatic HNSCC, pembrolizumab, an anti-programmed cell death protein 1 (PD-1) binding mAb, has recently gained FDA approval as a first-line treatment in R/M HNSCC (11, 12). In this regard, pembrolizumab alone and in combination with chemotherapy showed an improvement in overall survival (OS) compared to the previous standard regimen of cetuximab plus chemotherapy (11.6 and 13.0 vs. 10.7 months, respectively). However, response rates in the total population were only slightly improved among all groups (17% pembrolizumab alone; 36% pembrolizumab plus chemotherapy vs 36% cetuximab plus chemotherapy) (13, 14). As the immune system is a complex network of humoral and cellular interactions, the lack of drastic improvements in the antitumor response of pembrolizumab may be the result of alterations in the innate and adaptive immunity (15–17). Therefore, in-depth characterization of the TIME is required to identify possible combination partners that can alleviate immune evasive mechanisms. In addition, as tumors with a decreased immunogenicity are prone to selective survival (15), combined targeting may also result in improved responses through reinstitution of both anti-tumor-associated immune cells and/or reduction of the immunosuppressive TIME (18–21).

In this study, we characterized the immune infiltrative landscape of HNSCC by profiling the immune composition and the immune checkpoint ligand expression profile of HSNCC tumors. As such, we deliver insight into the prognostic impact of immune-related markers for HNSCC patients. Our results provide rationale for the development and guidance of new/ongoing clinical investigations within immuno-oncology for HNSCC.

## Material and methods

### Databases

RNA-seq data of HNSCC patient tumor tissue and neighboring healthy head and neck tissue included in The Cancer Genome Atlas (TCGA) firehose legacy dataset were obtained from The Cancer Immunome Atlas (TCIA) (hereon defined as ‘TCGA cohort’) (https://tcia.at/home). General patient history (gender, age, smoking, alcohol use), tumor characteristics (histology and TNM stage), and details of treatment (surgery, (neo-)adjuvant treatment) were last obtained on April 15, 2022 from cBioPortal (http://cbioportal.org). Survival time and outcome were obtained from The Human Protein Atlas (THPA) (www.proteinatlas.org). In addition, data on immune cell infiltration was obtained from TCIA as well. This TCGA cohort consisted of 530 patients including healthy neighboring tissue for 43 individuals. For tumor histology, the majority of tumor samples originated of tumors from the oral cavity (tongue, tonsil, palate, lip) (n=369). For tumor stages, most samples were derived from advanced stage patients (stage III and IVa origin, n=101 and 253, respectively). The TCGA dataset used in this study was evaluated for the presence of batch effects using the ‘TCGA Batch Effects Viewer’ (https://bioinformatics.mdanderson.org).

### Immune composition and differential gene expression analysis

The TCIA database platform was queried to obtain data on cellular composition based on CIBERSORT LM22, a versatile computational method for quantifying cell type fractions from bulk tissue gene expression profiles. Using the same TCGA cohort, the relative fraction of various immune cells was characterized in relation to clinicopathological parameters (22). Immune cells that were characterized consisted of B cells, DCs, M1 macrophages, M2 macrophages, monocytes, NK cells, neutrophils, CD4+ T cells, CD8+ T cells and regulatory T cells (Treg).

A literature search of the current trends in immuno-oncology was used to generate a panel of 45 markers for further investigation: 5’-nucleotidase (5NTE/CD73), B7-proteins (B7-H6, CD80, CD86, PD-L1, PD-L2, and CD276), carcinoembryonic antigen cell adhesion molecule 1 (CEACAM1), galectin 9 (Gal-9), high mobility group box 1 (HMGB1), immunoglobulin superfamily proteins (CD47, CD48 and VSIG-3), indoleamine 2,3-dioxygenase 1 (IDO1), interleukins (IL15-Rα and IL37), human leukocyte antigens (HLA-C, HLA-DP, HLA-DQ, HLA-DR and HLA-E), UL-16 binding proteins (ULBP1-6), MHC-class 1 chain related proteins (MICA/B), poliovirus related receptors (CD112, CD155/PVR), receptor tyrosine kinases (Axl, EGFR, MERTK, TYRO3), and tumor necrosis factor receptor superfamily members (CD30, CD40L, CD70, CD137L, FAS, HVEML, GITR-L, OX40L, RANKL and TRAIL). Gene expression data for this panel of markers was obtained from TCIA as Transcripts Per Million (TPM). Following selection of statistically significant differences (p<0.01), Log2FC (fold change) >1, or <0 was set as the cut-off to screen for differentially expressed genes (DEGs) between tumor and healthy tissue. Additionally, expression levels were compared between different tumor stages and primary sites.

### Patient selection and sampling

Formalin-fixed paraffin-embedded (FFPE) tissue samples of a total of 50 HNSCC patients diagnosed and/or treated at the Antwerp University Hospital (UZA) between 2008 and 2020 were collected in collaboration with the UZA Department of Pathology (hereon defined as ‘UZA cohort’). Patients who met the following criteria were included: age at diagnosis ≥18 year; primary tumor located in the larynx, (hypo- and oro-) pharynx or oral cavity; tumor histology confirmed as squamous cell carcinoma; tumors classified as clinical stage III or IV according to the American Joint Committee on Cancer staging manual (8th edition). General patient history (gender, age, smoking, alcohol use, white blood cell count (WBC), neutrophil to lymphocyte ratio (NLR)), tumor characteristics (histology and TNM stage, primary/relapsed tumor collection, details of treatment (surgery, (neo-)adjuvant treatment) and survival data were obtained from hospital registries and confirmed by governmental cancer death registries. Primary samples consisted of 43 resection specimens, while 7 were obtained from biopsy material. Available relapse samples consisted of 9 resection specimens and 1 biopsy sample. Resection samples were fixed in 4% formaldehyde for up to 32h, while biopsy samples were fixed for up to 12h. Samples were paraffin embedded on a routine basis. This retrospective study was approved by the Ethics Committee of the Antwerp University Hospital/University of Antwerp.

### Immunohistochemistry

Five-μm-thick sections were prepared from FFPE tissue blocks. Sections were baked for 1h at 60°C prior to staining. Immunohistochemistry (IHC) was optimized for the Dako Autostainer Link 48 (Agilent Technologies, Santa Clara, CA) in combination with the Envision Flex DAB+ and HRP Magenta Chromogen system (Agilent), with minor alterations to the manufacturer’s datasheet (23, 24). The following antibodies were used: anti-CD4 (clone SP35, RTU, Ventana), anti-CD8 (clone SP57, RTU, Ventana), FoxP3 (clone 236A/E7, Abcam), NKp46 (clone 195314, Bio-Techne), EGFR (clone H11, Cell Signaling Technology), CD47 (clone B6H12.2, Thermo-Fisher), CD70 (polyclonal, Thermo-Fisher), CD73 (clone D7F9A, Cell Signaling Technology), CD155 (clone D8A5G, Cell Signaling Technology), CD276 (clone D9M2L, Cell Signaling Technology), HLA-E (MEM−E/02, LifeSpan BioSciences) and PD-L1 (clone E1L3N, Cell Signaling Technology). All tissue slides were counterstained with hematoxylin (Agilent) and an additional hematoxylin and eosin (HE) staining was provided for each patient. Sections were washed in Dako wash buffer (Agilent), dehydrated in xylene, graded alcohol, mounted with Quick-D Mounting Medium (Klinipath) and coverslipped. Placenta tissue was included as a positive control for PD-L1 while tonsil tissue was used for all other markers. All slides were scored by a pathologist, as described previously (24). Briefly, the tumor immune phenotype of each patient was defined using HE-slides and classified as inflamed (‘hot’), non-inflamed (‘deserted’) or immune-excluded (‘excluded’). Subsets of lymphoid cells (CD4/CD8/FoxP3/NKp46 positivity) were scored both intratumorally and in the tumor stroma. Percentages were classified as: 0 (<1%); I (1–5%); II (6–10%); III (>10%). Immune checkpoint ligands were scored according to tumor proportion scores (TPS) in the main tumor bulk and categorized into following five groups: 0 (<1%); I (1–10%); II (11–29%); III (30–60%): IV (>60%). Additionally, PD-L1 was also scored for the combined positive score (CPS) as is currently being used in determining PD-1 treatment eligibility (11).

### Survival analysis

The prognostic impact of the clinicopathological parameters and expression data of the TCGA and UZA cohorts were investigated using univariate Cox proportional hazards tests. Significant parameters were further included in multivariate models. Overall survival (OS) was calculated from the day the cancer was diagnosed to the date of death (all causes) or the end of data recording (April 15, 2022). Risk ratios were used to determine the prognostic impact. Survival curves of DEGs were plotted in Kaplan-Meier survival plots using GraphPad Prism (version 9.0, GraphPad Software, San Diego, CA, USA). TCGA data were grouped in either ‘High’ or ‘Low’ groups based on cut-off values either calculated using ‘Cutoff Finder’ online tool (immune composition) or obtained from THPA (Checkpoint ligand expression) (25). Survival curves for IHC analyses were grouped based on the IHC-scoring categories.

### Statistical analysis

TCGA and IHC analyses were performed using JMP^®^ Pro (version 15.1.0, SAS institute Inc. Cary, NC). Univariate comparisons between clinicopathological parameters and expression data were performed either parametrically (for TCGA data) using T-test (level=2) or One way ANOVA (level>2) or non-parametrically (IHC) using the Mann-Whitney U (level=2) or Kruskal-Wallis (level>2) tests. Correlation between immune cell compositions and immune checkpoint ligands were assessed parametrically (Pearson) for TCGA gene expression and non-parametrically for IHC positivity (Spearman). Univariate cox proportional hazards models were used to determine the impact of clinicopathological parameters on survival. To correct for the influence of these parameters on patient prognosis when considering the impact of immune cells and immune checkpoint ligand expression, univariately significant clinicopathological parameters were added in a multivariate cox proportional hazards model with and without the IHC staining data of different markers. The likelihood that IHC scoring provided a better fit in this model was based on Chi2 distribution and probability calculation. Models that provided a significant probability were used to investigate risk ratios. Statistical significance was defined as p<0.05, with p-values corrected for multiple testing using the false discovery rate, unless defined otherwise.

## Results

### Clinical covariates affect the immune composition of the TCGA cohort

**Figure 1:**
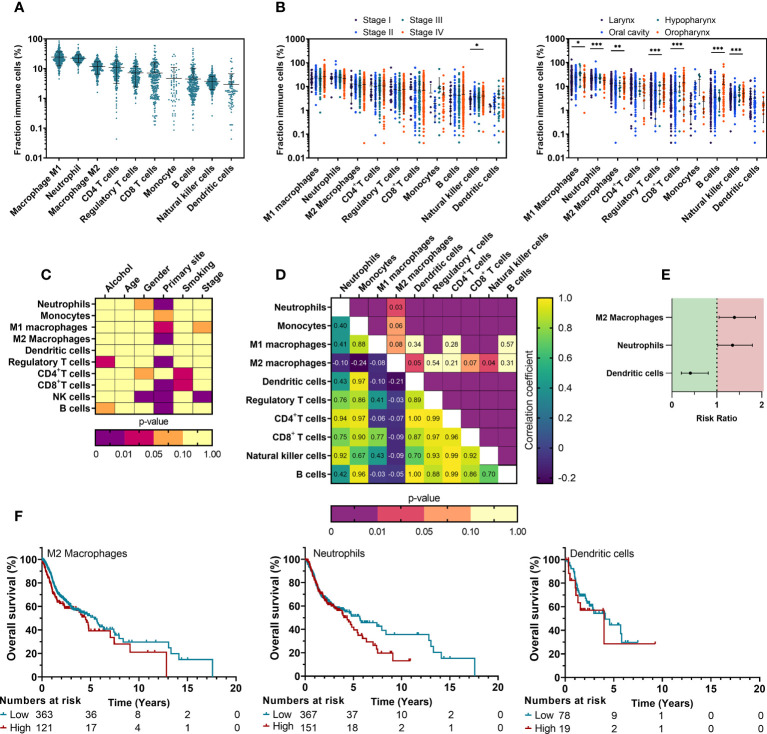
Composition of TIICs in HNSCC depends on clinical parameters and impacts prognosis of HNSCC patients. **(A)** Distribution of different TIICs across tumor stages and primary sites was obtained from the TCIA database using CIBERSORT computing. **(B)** Associations between the fractions of characterized TIICs and clinicopathological parameters were univariately analyzed using T-test (level=2) or One way ANOVA (level>2). **(C)** Changes in NK cell relative fractions are shown for clinicopathological parameters that showed statistical significance. **(D)** Correlations between fractions of all TIICs were investigated to assess possible associations using a Pearson’s correlation matrix. **(E)** A multivariate Cox proportional hazards model was used to identify the prognostic effect. **(F)** Kaplan-Meier survival curves were generated for TIICs that showed significance from multivariate Cox regression. TIICs were classified as ‘High’ or ‘Low’ using ROC fitment (CutoffFinder). Statistical significance was defined as p<0.05.

To better understand the immune signature of HNSCC, we initiated our investigation by analyzing the TCGA cohort. The clinicopathological characteristics of this cohort are summarized in **Table 1**. As it has been stated in literature that the presence of tumor infiltrating immune cells (TIICs) is correlated with clinical outcome (26), quantifying the fraction of TIICs could serve as a prognostic biomarker for anti-cancer treatments (27). With that in mind, we characterized the TIICs in tissue samples of the TCGA cohort and determined whether the presence of TIICs could be correlated with survival. Data regarding immune cell fractions was obtained through TCIA, using CIBERSORT, to estimate the fraction of 10 different immune cell types in this cohort. HNSCC tumors had an abundance of tumor-associated macrophages (36.8 ± 11.9%), neutrophils (22.8 ± 9.78%) and CD4+ T cells (10.5 ± 8.04%), Tregs (7.50 ± 7.15%) and CD8+ T cells (6.84 ± 9.18%) whereas B cells (4.46 ± 6.88%), NK cells (3.76 ± 2.30%) and DCs (2.98 ± 4.04%) were less present (**Figure 1A**). Furthermore, univariate analysis of TIICs in relation with clinicopathological parameters (**Figure 1C**) revealed NK cell percentages were significantly higher in male patients (p<0.001) (**Figure S1**). Higher tumor stages were also associated with increased NK cells (p=0.030) (**Figures 1B**, **S1**). Primary tumor site showed a clear association with nearly all immune cell types, indicating a differential immune composition in different histological subtypes (**Figures 1B**, **S1**). Although a large portion of patients had missing data regarding their smoking behavior, we observed an increased abundance of CD4+ (p=0.010) and CD8+ T cells (p=0.021) in patients with a smoking history, while excessive alcohol consumption was associated with an increased Treg fraction (p=0.048) (**Figures 1C**, **S1**). TIICs showed a moderate-to-strong positive correlation between each other, except for macrophages: M1 macrophages were only correlated with CD8+ T cells, while M2 macrophages negatively correlated with DCs (**Figure 1D**). Next, the prognostic impact of TIICs was investigated. Univariate Cox regression did not reveal a significant effect of clinicopathological parameters on OS. Therefore, a cox regression model without any corrections for the TIIC fraction was used to assess survival (**Table S2**). This model revealed a significantly worse OS with an increased number of M2 macrophages (RR: 1.39 [1.05–1.86], p=0.023), and neutrophils (RR: 1.35 [1.02–1.79], p=0.031). In contrast, infiltration of DCs was linked with a better OS (RR: 0.41 [0.21–0.81], p=0.010) (**Figure 1E**). All other TIICs showed no prognostic effect. Kaplan-Meier survival curves of M2 macrophages, neutrophils and DCs were generated (**Figure 1F**). Taken together, this data indicates that the TIME for our TCGA cohort of HNSCC patients is substantially infiltrated by various immune cells of the innate and adaptive immune system and that among them the increased presence of M2 macrophages, neutrophils and DCs may have the largest prognostic impact.

**Table 1:** Clinicopathological characteristics of the TCGA and UZA patient cohorts.

Patient characteristics	TCGA tumor cohort (n=530)	TCGA healthy cohort(n=43)	UZA cohort(n=50)
Gender (%)			
Male	387 (73)	29 (50)	38 (76)
Female	143 (27)	29 (50)	12 (24)
Age (years)			
Mean ± SD	61 ± 12	62 ± 14	72 ± 10
Range	19 – 90	29 – 87	53 – 90
Smoking status (%)			
Current smoker	178 (33.6)	11 (25.6)	20 (40)
Former smoker	217 (40.9)	20 (46.5)	8 (16)
Never smoked	122 (23.0)	11 (25.6)	2 (4)
Not indicated	13 (2.5)	1 (2.3)	20 (40)
Alcohol (%)			
Current drinker	168 (31.7)	15 (34.9)	25 (50)
Ex drinker	–	–	2 (4)
Non-drinker	57 (10.8)	4 (9.3)	1 (2)
Not indicated	305 (57.5)	24 (55.8)	22 (44)
Tissue source (%)			
Primary resection	–	–	43 (86)
Primary biopsy	–	–	7 (14)
Relapsed resection	–	–	9 (90)
Relapsed biopsy	–	–	1 (10)
Not indicated	530 (100)	43 (100)	
Primary tumor site (%)			
Larynx	117 (22.1)	11 (25.6)	17 (34.0)
Larynx	117 (22.1)	11 (25.6)	12 (24.0)
Glottis	–	–	5 (10.0)
Hypopharynx	10 (1.9)	–	–
Oral Cavity	348 (65.7)	32 (74.4)	28 (56.0)
Alveolar ridge	18 (3.4)	–	–
Buccal mucosa	23 (4.3)	–	1 (2.0)
Floor of mouth	64 (12.1)	3 (7.0)	7 (14.0)
Hard palate	7 (1.3)	–	–
Gum	–	–	2 (4.0)
Lip	3 (0.6)	–	–
Oral cavity	73 (13.8)	14 (32.6)	2 (4.0)
Oral tongue	133 (25.1)	13 (30.2)	16 (32.0)
Oropharynx	55 (10.4)	–	5 (10.0)
Base of tongue	27 (5.1)	2 (4.7)	–
Oropharynx	9 (1.7)	–	2 (4.0)
Tonsil	46 (8.7)	–	3 (6.0)
HPV status HPV/p16 positive	0 (0)	0 (0)	0 (0)
Staging (%)			
I	21 (4.0)	–	–
II	99 (18.7)	–	–
III	107 (20.2)	–	14 (28)
IVa	271 (51.1)	–	33 (66)
IVb	11 (2.1)	–	3 (6)
IVc	7 (1.3)	–	–
Not indicated	14 (2.6)	–	–
Treatment (%)			
Neo-adjuvant regimens	10 (1.9)	–	8 (16)
Chemotherapy	–	–	2 (4)
Radiotherapy	–	–	5 (10)
Chemoradiation	–	–	1 (2)
Surgery	–	–	50 (100)
Adjuvant regimens	192 (36.2)	–	39 (78)
Radiotherapy	–	–	12 (24)
Chemoradiation	–	–	21 (42)
Cetuximab	–	–	4 (8)
Immunotherapy	–	–	2 (4)
White blood cell count (10^9^ cells/l)		–	
Mean ± SD	–	–	8.95 ± 4.12
Median (range)	–	–	8.28 (2.3 – 23.4)
Neutrophil/lymphocyte ratio		–	
Mean ± SD	–	–	9.83 ± 10.13
Median (range)	–	–	7.66 (0.73 – 71.7)
Survival (%)		–	
Alive	304 (57.6)	–	22 (44)
Dead	224 (42.4)	–	28 (56)
Not indicated	2 (0.4)	–	–
Follow-up			
Mean ± SE	3.93 ± 0.20		5.24 ± 0.48
Median	2.87 (2.60 – 3.20)		5.52 (3.41 – 8.81)
Overall survival (years)		–	
Mean ± SE	6.43 ± 0.49	–	6.99 ± 0.71
Median (95% CI)	4.70 (3.83 – 5.65)	–	4.69 (3.59 – 9.64)
Progression free survival (years)		–	
Mean ± SD	3.37 ± 0.16	–	3.37 ± 0.49
Median (range)	2.80 (2.49 – 3.08)	–	1.87 (1.01 – 2.57)

SD, Standard deviation; SE, Standard error; CI, Confidence interval.

### Immune checkpoint ligands show differential expression with prognostic relevance in the TCGA cohort

**Figure 2:**
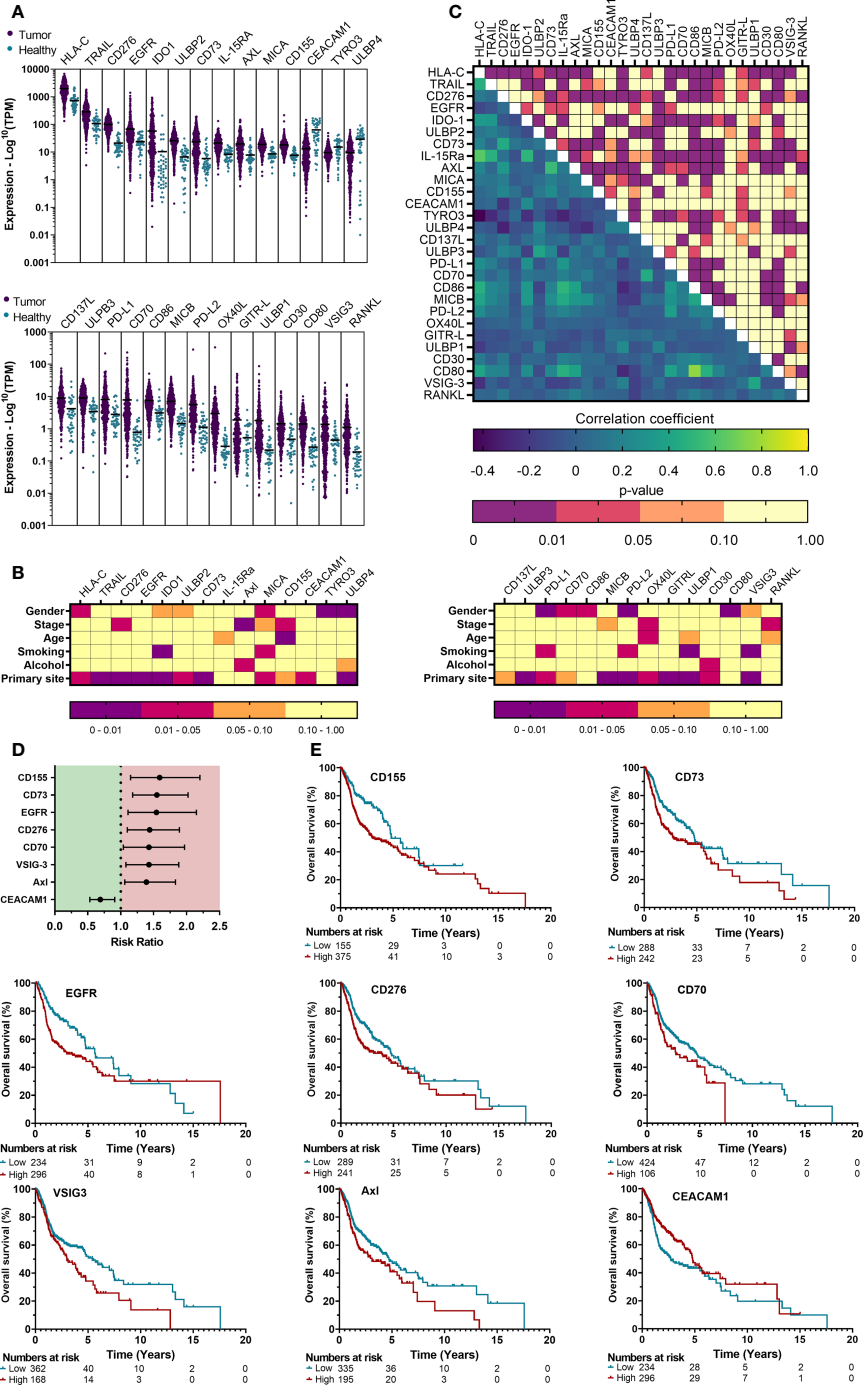
Gene expression profile analysis of the TCGA cohort reveals differentially expressed immune checkpoint ligands that are associated with clinicopathological parameters and prognosis. **(A)** mRNA expression of HNSCC patient tumor tissue represented the logarithmic TPM (Transcripts per million) was compared with neighboring healthy head and neck tissue to identify differentially expressed genes (DEGs), defined as Log2FC>1 or Log2FC<0 with p<0.01. **(B)** The associations between gene expression and various clinicopathological parameters were univariately analyzed using T-test (level=2) or One way ANOVA (level>2). **(C)** Possible correlations in gene expression among all checkpoint ligands was investigated through a Pearson’s correlation matrix. **(D)** Median survival was determined, and survival analysis was performed using multivariate Cox proportional hazards. **(E)** Kaplan-Meier survival curves were generated for TIICs that showed significance from multivariate Cox regression. Cut-off values to determine “High” vs “Low” expression were obtained from THPA. Kaplan-Meier survival curves were generated for ligands with significant prognostic effects. Statistical significance was defined as p<0.05.

In order to identify immune checkpoint ligands that were most differentially expressed in a large population of patients, we obtained expression data of 530 HSNCC patients and head and neck tissue from 43 healthy individuals. Differential expression between ‘Tumor’ vs ‘Healthy’ data (**Figure 2A**, **Table S3**) identified 28 DEGs in HNSCC tumor samples. Of these, 25 genes were significantly upregulated (Log2FC>1, p<0.01), while 3 showed significant downregulation (Log2FC<0, p<0.01) in tumor samples. Tumor staging, primary tumor sites and other clinicopathological parameters were univariately analyzed to observe associations with gene expression (**Figures 2B**, **S2**). Alcohol consumption (3/28) was least associated with DEGs. In contrast, primary tumor site (19/27) was most associated with DEGs. Correlating the expression of all immune checkpoint ligands suggested possible associations within the HLA genes and TNF superfamily (CD30, CD40L, CD70, CD137L, GITR-L, HVEML, OX40L, RANKL, FAS, TRAIL, RANKL) (**Figure 2C**). Among the DEGs, a moderate-to-strong correlation was only observed between CD80 – CD86 (r=0.760), HLA-C – IL-15Ra (r=0.627) and AXL – CD73 (r=0.534). Next, we investigated whether the identified DEGs are also associated with patient prognosis. As univariate Cox regression of clinicopathological parameters did not indicate a significant effect on patient prognosis (**Table S1**), a univariate Cox regression was used for survival analysis. Increased expression of CD155 (RR: 1.59 [1.15–2.2], p=0.005), CD73 (RR: 1.55 [1.18–2.02], p=0.002), EGFR (RR: 1.54 [1.11–2.15], p=0.011), CD276 (RR: 1.44 [1.10–1.89], p=0.007), CD70 (RR: 1.43 [1.04–1.97], p=0.028), VSIG-3 (RR: 1.43 [1.08–1.88], p=0.012) and Axl (RR:1.39 [1.06-1.83], p=0.017), were associated with a worse prognosis, while CEACAM1 (RR: 0.69 [0.53–0.91], p=0.008) showed a significantly better outcome with higher expression (**Table S2**, **Figure 2D**). Gene expression of these 8 prognostic markers were classified as either ‘High’ or ‘Low’ based on cutoff values provided by THPA and visualized by Kaplan-Meier survival curves (**Figure 2E**). Taken together, these observations suggest that several immune checkpoint ligands might play a role in the TIME of HNSCC and could be regarded as interesting markers for further evaluation in the context of antitumor therapies.

### Clinicopathological characteristics of the UZA cohort

To further explore the relevance of these DEGs with the most prognostic impact on a tissue level, we selected a cohort of 50 stage III and IV HNSCC patients treated in the UZA. The clinicopathological characteristics of this cohort are summarized in **Table 1**. Patients who had additional matched biopsies of the primary tumor following relapse were also included (n=10). Patients were predominantly male (76%) and had a median age of 71 years (range, 53–90). At the time of last follow-up (April 15, 2022), 44% of patients were still alive.

### Immune composition of the UZA cohort correlates with patient outcome

**Figure 3:**
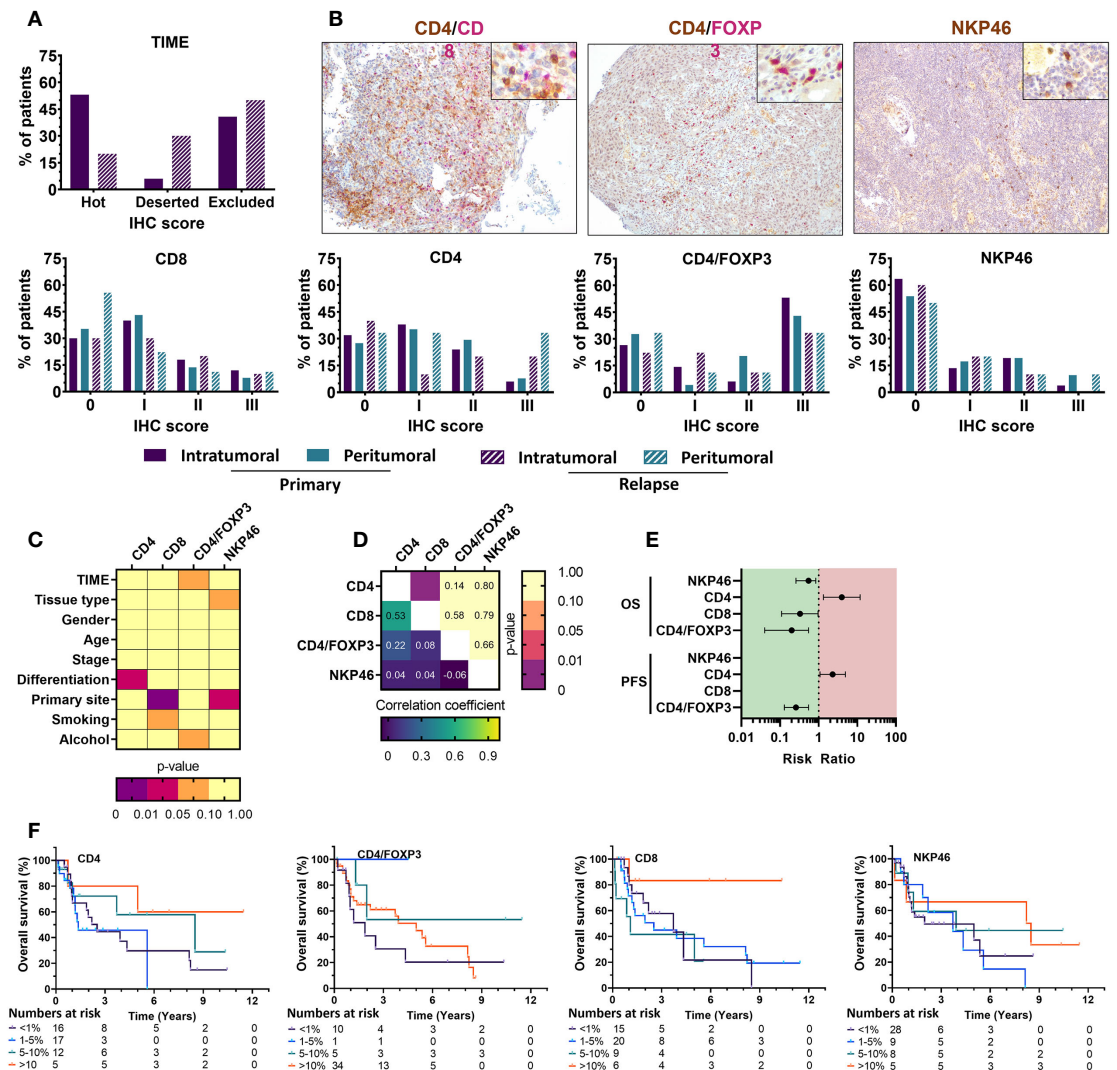
Immunohistochemical staining of immune cell markers in FFPE tissue from primary and relapsed HNSCC patients shows association with clinicopathological parameters and prognostic relevance with overall survival. **(A)** The tumor immune phenotype of HNSCC patients was evaluated using HE stains an together with various immune cell markers showed distinct positivity among HNSCC patients. Intratumoral immune cell infiltration was compared with the presence of stromal immune cell populations. Primary and relapsed tumor samples were compared in their composition as well. Positivity was defined as 0 (<1%); I (1–5%); II (6–10%); III (>10%) **(B)** Representative images of CD4(brown)/CD8(pink) and CD4(brown)/FoxP3(pink) dual staining together with NKp46(brown) single staining of HNSCC tissue shown at 100x, with an inlet at 400x magnification. **(C)** Associations between the immunohistochemical scoring and various clinicopathological parameters was univariately analyzed using Mann-Whitney U (level=2) or Kruskal-Wallis statistical (level>2) tests. **(D)** Correlations between different immunohistochemical markers was investigated through a Spearman rank correlation. **(E)** Median survival was determined, and survival analysis was performed using multivariate Cox proportional hazards. **(F)** Kaplan-Meier survival curves were generated for tumor-infiltrating immune cells with statistically significant prognostic effects. Statistical significance defined as p<0.05.

Within the TIME, different immune cells contribute differently to/against tumor progression. However, a great emphasis has been established for the presence of tumor infiltrating lymphocytes during therapy development and optimization. Evidence indicates that when TILs are present in the tumor as dense aggregates of activated immune cells, tumor prognosis and responses to therapy are favorable (28). Therefore, all samples were analyzed for the spatial distribution of immune cells in the TIME. The majority of primary tumor samples had either a high degree of immune infiltration (53.1%) or showed an absence of noticeable immune cells (‘deserted’) in the tumor and its periphery (40.8%). Exclusion of immune cells at the tumor periphery were low (6.1%). For tissue slides of relapsed tumor samples, a ‘deserted’ TIME (50%) was primarily observed, while accumulation near the tumor periphery (30%) or infiltration in the tumor (20%) were less present (**Table 2**). Beyond that, the presence of intratumoral and peritumoral lymphocytes (**Table 2**) were investigated. Tissue sections were stained for four lymphocyte subsets: CD4+ T cells, CD8+ T cells, CD4+FoxP3+ regulatory T cells and NKp46+ NK cells (**Table 2**, **Figure 3B**). Tumor infiltration by lymphocytes was observable throughout the tumor bulk as well as hot spots of lymphoid aggregates.

**Table 2:** Expression of immune cell markers in FFPE tissue sections of treatment naïve (prim) or relapsed (relap) HNSCC patients.

Lymphocyte infiltration	CD4				CD8				CD4/FoxP3				NKp46			
	Intratumor		Peritumor		Intratumor		Peritumor		Intratumor		Peritumor		Intratumor		Peritumor	
	Pr	Re	Pr	Re	Pr	Re	Pr	Re	Pr	Re	Pr	Re	Pr	Re	Pr	Re
Total % positive	68.0	50.0	72.5	66.7	70.0	60.0	64.7	44.4	73.5	75.0	67.3	62.5	36.5	33.3	46.2	44.4
0 (<1%)	32.0	40.0	27.5	33.3	30.0	30.0	35.3	55.6	26.5	25.0	32.7	37.5	63.5	66.7	53.8	55.6
I (1 – 5%)	38.0	10.0	35.3	33.3	40.0	30.0	43.1	22.2	14.3	25.0	4.1	12.5	13.5	22.2	17.3	22.2
II (6 – 10%)	24.0	20.0	29.4	0.0	18.0	20.0	13.7	11.1	6.1	12.5	20.4	12.5	19.2	11.1	19.2	11.1
III (>10%)	6.0	20.0	7.8	33.3	12.0	10.0	7.8	11.1	53.1	37.5	42.9	37.5	3.8	0.0	9.6	11.1
Average ratio with CD8	44.3	43.8	58.9	66.7	–	–	–	–	28.1	34.9	37.2	36.5	–	–	–	–
	**TIME**															
	Pr	Re														
Hot	53.1	20.0														
Excluded	6.1	30.0														
Deserted	40.8	50.0														

Pr, Primary; Re, Relapsed.

The majority of patient tissue samples (primary: 68%, relapsed: 50%) were positive for intratumoral CD4+ staining with the highest percentage of CD4+ T cells infiltration being 12.3% (median: 2.00%). Although not statistically significant, the percentage of patients with CD4+ T cells in peritumoral stroma was greater (primary: 72.5%, relapsed: 66.7%) and reached a max of 21% (median: 2.62%) of cells in the peritumoral stroma (p=0.298) compared to intratumoral CD4+ T cells. CD8+ T cells were similarly present in the majority of intratumoral tissue parts (primary: 70%, relapsed: 60%) while peritumoral CD8 percentages in relapsed tissue samples were present in less than 50% of patients (primary: 64%, relapsed: 44%). The highest percentage of CD8 positivity was 21% (median: 1.75%) intratumorally and 20% (median: 1.6%) in the tumor stroma. Although our observations suggested a slight dominance of CD8+ T cells intratumorally (ratio CD4/CD8: 43.7%) and CD4+ T cells in the peritumoral stroma (ratio CD4/CD8: 58.9%), these observations were not statistically significant (p=0.296), nor was the difference between primary and relapsed percentages (p=0.059). Regulatory T cells, identified as CD4+FoxP3+ cells, were observed to a greater extent intratumorally (primary; 73.5%, relapsed; 75.0%) than found in peritumoral tissue (primary; 67.3%, relapsed; 62.5%). The highest percentage of positivity was 12% intratumorally (median: 0.6%) and 14% peritumorally (median: 0.5%). Statistical evidence to suggest a significant difference between tumor and peritumoral stroma (p=0.636) and primary or relapsed tissue samples (p=0.155) was not present. Lastly, NKp46+ NK cells were detected in less than 50% of patients both intratumorally (primary: 36.5%, relapsed: 33.3%) and in peritumoral stroma (primary: 46.2%, relapsed: 44.4%). NK cells showed a greater percentage of positivity peritumorally of 10% (median: 1.37%), while inside the tumor it only accounted for 5% of cells (median: 0.5%) (**Table 2**, **Figures 3A, B**). Importantly, statistical testing did not confirm the possible differences between intratumoral and peritumoral positivity (p=0.218) nor between primary and relapsed tissue (p>0.276). Clinicopathological parameters were univariately analyzed for associations with immune cell presence (**Figure 3C**, **S3**). Tumor differentiation showed a significant association with increased CD4 positivity (p=0.029) while primary tumor site showed the differential presence of CD8+ (p=0.001) and NKp46+ (p=0.015) positivity, respectively. Furthermore, total WBC counts significantly but weakly correlated with NKp46 staining (r=0.220; p=0.031), while NLR ratio negatively correlated with CD8 staining (r=-0.246; p=0.034). Correlation of the immune cell markers with each other revealed a positive correlation between CD4 and CD8 staining (r=0.48; p<0.001) (**Figure 3D**). Univariate Cox regression indicated a significant effect of primary tumor site (p=0.017), age at diagnosis (p<0.001) smoking (p<0.001) on survival (**Table S1**). These were all corrected for in a multivariate model. No clinicopathological parameters significantly affected progression free survival (PFS) and was therefore ran univariately.

Using multivariate Cox regression, we found a significant association between the presence of tumor-infiltrating CD4+ T cells and worse OS (RR: 3.98 [1.34–11.82], p=0.013) but not stromal CD4+ T cells. CD8+ T cell infiltration was marked by an improved OS (RR: 0.31 [0.11–0.97], p=0.044), while stromal CD8+ T cells did not show significance. Intratumoral CD4+FoxP3+ cells similarly associated with improved OS (RR: 0.20 [0.04–0.55], p=0.016), while stromal CD4+FoxP3+ cells indicated a worse OS (RR: 3.05 [1.03–9.06], p=0.032). Tumor infiltrating NKp46+ cells also adhered to a better outcome with increased infiltration (RR: 0.55 [0.26–0.84], p=0.037), while stromal NKp46+ cells showed no statistical correlation (**Table S4**, **Figure 3E**). These effects were represented by Kaplan-Meier survival plots as well (**Figure 3F**). PFS was somewhat similar, with intratumoral CD4+ T cells showing worse PFS (RR: 2.31 [1.09–4.93], p=0.012), while CD4/FoxP3 positivity was marked by an improved PFS (RR: 0.26 [0.13–0.55], p=0.005). In contrast, neither CD8 nor NKp46 positivity showed significant effects (**Figure 3E**). Taken together, these results show that despite CD4+/FoxP3+ cells making up a large portion of tumor infiltrating lymphocytes within the TIME of our UZA cohort, the presence of intratumoral cytotoxic lymphocytes such as CD8+ T cells and NKp46+ NK cells was overall much stronger and results in a better patient outcome.

### Immune checkpoint ligand expression of the UZA cohort reveals several targetable proteins

**Figure 4:**
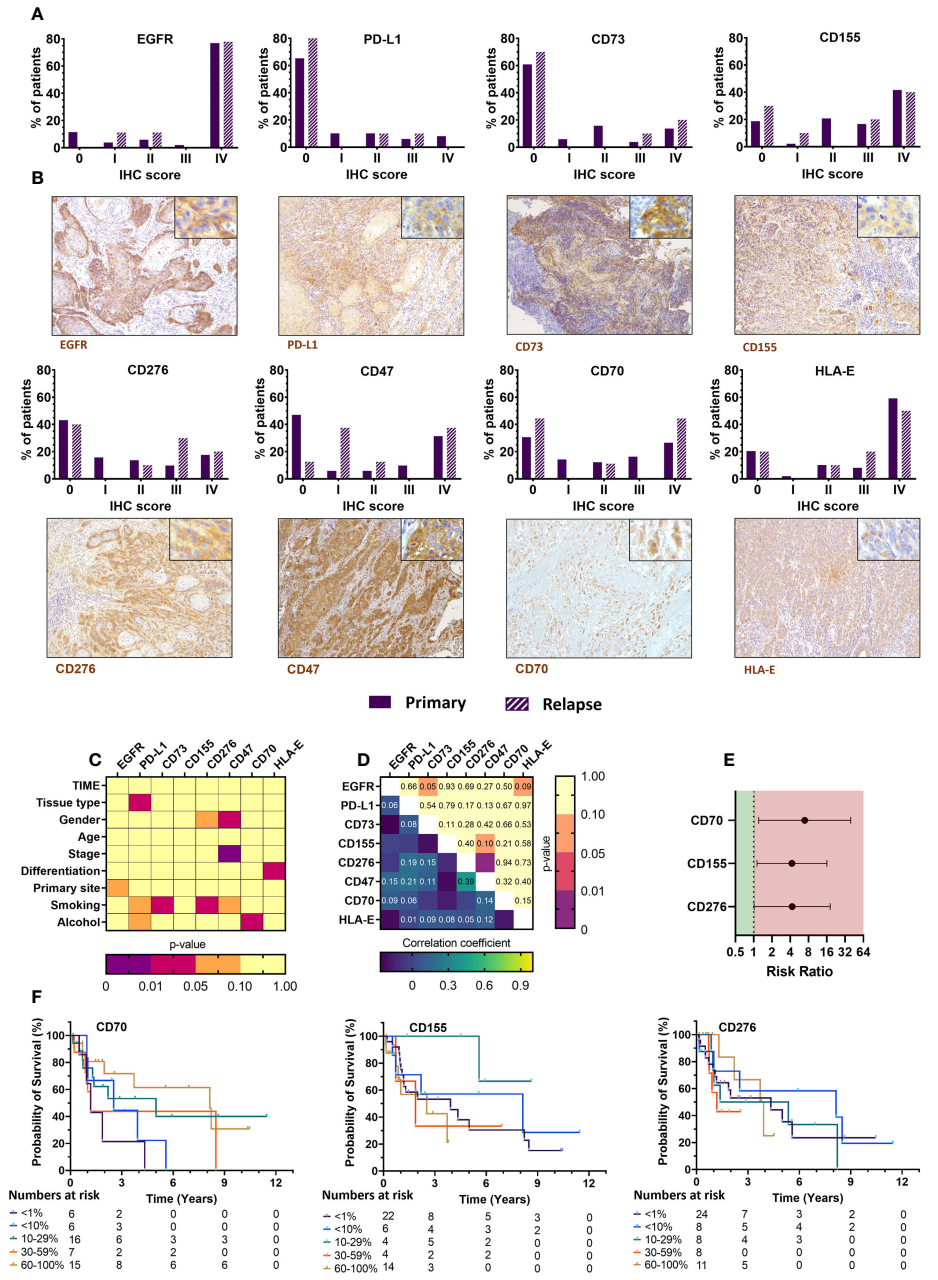
Immunohistochemical staining of immune checkpoint ligand markers in FFPE tissue from primary and relapsed HNSCC patients shows association with clinicopathological parameters and prognostic relevance with overall survival. **(A)** Intratumoral expression patterns of various immune checkpoint ligands differed greatly among HNSCC patients. Primary tumor samples were compared with relapsed tumor samples as well. Positivity was defined as 0 (<1%), I (1-10%), II (11–29%), III (30–59%) or IV (60–100%). **(B)** Representative images of EGFR, PD-L1, CD73, CD155, CD276, CD47, CD70, and HLA-E (brown) stainings of HNSCC tissue were shown at 100x, with an inlet at 400x magnification. **(C)** Associations between the immunohistochemical scoring and various clinicopathological parameters were univariately analyzed using Mann-Whitney U (level=2) or Kruskal-Wallis statistical (level>2) tests. **(D)** Correlations between different immune checkpoint ligands was investigated through Spearman rank correlation. **(E)** Median survival was determined, and survival analysis was performed using multivariate Cox proportional hazards. **(F)** Kaplan-Meier survival curves were generated for tumor-infiltrating immune cells with statistically significant prognostic effects. Statistical significance defined as p<0.05.

The current treatment landscape of HNSCC involves the use of cetuximab (anti-EGFR) and pembrolizumab (anti-PD-1) as first line treatments. Since EGFR overexpression is frequently observed (>80%) in HNSCC patients and plays a pivotal role in the onset of HNSCC (29, 30), we assessed whether our sample set contained sufficient EGFR-negative tumors samples, in order to compare their immune composition and immune phenotype to EGFR-positive tumors. Primary samples were in that regard 88.5% EGFR-positive while relapsed samples were all EGFR-positive (**Table 3**, **Figure 4A**), limiting the ability to analyze EGFR on relapsed samples. In addition, tissue slides were also stained for PD-L1, as patient eligibility for pembrolizumab treatment is dependent on a PD-L1 CPS of at least 1 (31–33). We scored PD-L1 expression using both CPS and TPS, the latter which was used to maintain a uniform scoring. Based on the TPS data, the majority of patients (65.3%) had a PD-L1 expression lower than 1% while the combined PD-L1 expression on tumor and immune cells was greater than 1% for most patients. Furthermore, we expanded our investigation by also staining tissue sections for CD70, CD73, CD155 and CD276, as inhibitory immune checkpoint ligands associated with a negative prognosis as identified from our TCGA analysis. There were considerable differences in the tumor expression of these markers with CD155 showing highest expression in the majority of samples (81.3%). CD70 was expression was mostly either very high (26.5%) or very low (30.6%). CD276, was diffusely present in 56.9% of patients while CD73 expression was undetectable in most (60.8%). In addition, tissue slides were stained for CD47 and HLA-E as well, considering that interaction of these markers with their respective receptors SIRPα and NKG2A has gained increasing interest in HNSCC research as alternative immune checkpoint pathways (34, 35) (**Figures 4A, B**). CD47 expression was somewhat similar CD70, with patients either showing high or low expression. HLA-E, clearly showed high expression in most patients (59.2%) (**Table 3**, **Figure 4A**). Univariate analysis of checkpoint ligands with clinicopathological parameters was performed in order to identify relevant associations (**Figures 4C**, **S4**). No association of EGFR and CD155 with any clinicopathological parameters was observed. PD-L1 expression was higher in primary samples compared to relapsed samples (p=0.047). Non-smokers showed significantly higher CD73 and CD276 expressions, but this was likely biased by the limited sample size of this group (n=2). CD47 expression was higher in females (p=0.047) and in more advanced TNM stages (p=0.002). We observed that CD70 expression was increased in non-drinking patients (p=0.026). However, the limited availability of patient information regarding their alcohol consumption translated into small sample sizes of each group. Lastly, HLA-E expression was associated with better differentiated tumors (p=0.04). Correlation analyses between different markers showed no correlation with EGFR expression, except for CD276 and CD47 (r=0.393; p=0.003) (**Figure 4D**). Survival analyses showed that (i) CD70 (RR: 6.94 [1.21–39.68], p=0.029); (ii) CD276 (RR: 4.31 [1.02–18.24], p=0.047); (iii) CD155 (RR: 4.27 [1.14–16.08], p=0.032) were associated with a worse survival. These prognostic effects were represented by Kaplan-Meier survival plots as well (**Figure 4E**) Finally, patients with high CD276 (RR: 4.92 [1.03–23.25], p=0.031), or PD-L1 (RR: 3.69 [1.31–10.35], p=0.009) expression were more likely to have shorter PFS. Taken together, the negative prognostic impact of PD-L1 expression on patient outcome highlights its clinical importance. In that sense, the negative prognostic impact of CD155 and CD276 could also be of equal importance and thus warrants further investigation as potential novel immune checkpoint therapies.

**Table 3:** Expression of immune checkpoint markers in FFPE tissue sections of treatment naive or relapsed HNSCC patients.

Tumor checkpoint ligand positivity	CD47		CD70		CD73		CD155		CD276	
	Pr	Re	Pr	Re	Pr	Re	Pr	Re	Pr	Re
Total % positive	52.9	87.5	69.4	55.6	39.2	30.0	81.3	70.0	56.9	62.5
0 (<1%)	47.1	12.5	30.6	44.4	60.8	70.0	18.8	30.0	43.1	40.0
I (1 – 10%)	5.9	37.5	14.3	0.0	5.9	0.0	2.1	10.0	15.7	0.0
II (11 – 29%)	5.9	12.5	12.2	11.1	15.7	0.0	20.8	0.0	13.7	10.0
III (30 – 59%)	9.8	0.0	16.3	0.0	3.9	10.0	16.7	20.0	9.8	30.0
IV (60 – 100%)	31.4	37.5	26.5	44.4	13.7	20.0	41.7	40.0	17.6	20.0
	**EGFR**		**HLA-E**		**PD-L1**					
					TPS		CPS			
	Pr	Re	Pr	Re	Pr	Re	Pr	Re		
Total % positive	88.5	100.0	79.6	80.0	34.7	20.0	66.0	40.0	Total % positive	
0 (<1%)	11.5	0.0	20.4	20.0	65.3	80.0	34.0	60.0	0 (<1%)	
I (1 – 10%)	3.8	11.1	2.0	0.0	10.2	0.0	44.7	30.0	I (1-20%)	
II (11 – 29%)	5.8	11.1	10.2	10.0	10.2	10.0	12.8	10.0	II (>20%)	
III (30 – 59%)	1.9	0.0	8.2	20.0	6.1	10.0	8.5	0.0	III (>50%)	
IV (60 – 100%)	76.9	77.8	59.2	50.0	8.2	0	–	–		

CPS, Combined positive score; Pr, Primary; Re, Relapsed; TPS, Tumor proportion score.

### Immune profiling of the TCGA and UZA cohorts based on their clinicopathological parameters, expression data and survival

**Figure 5:**
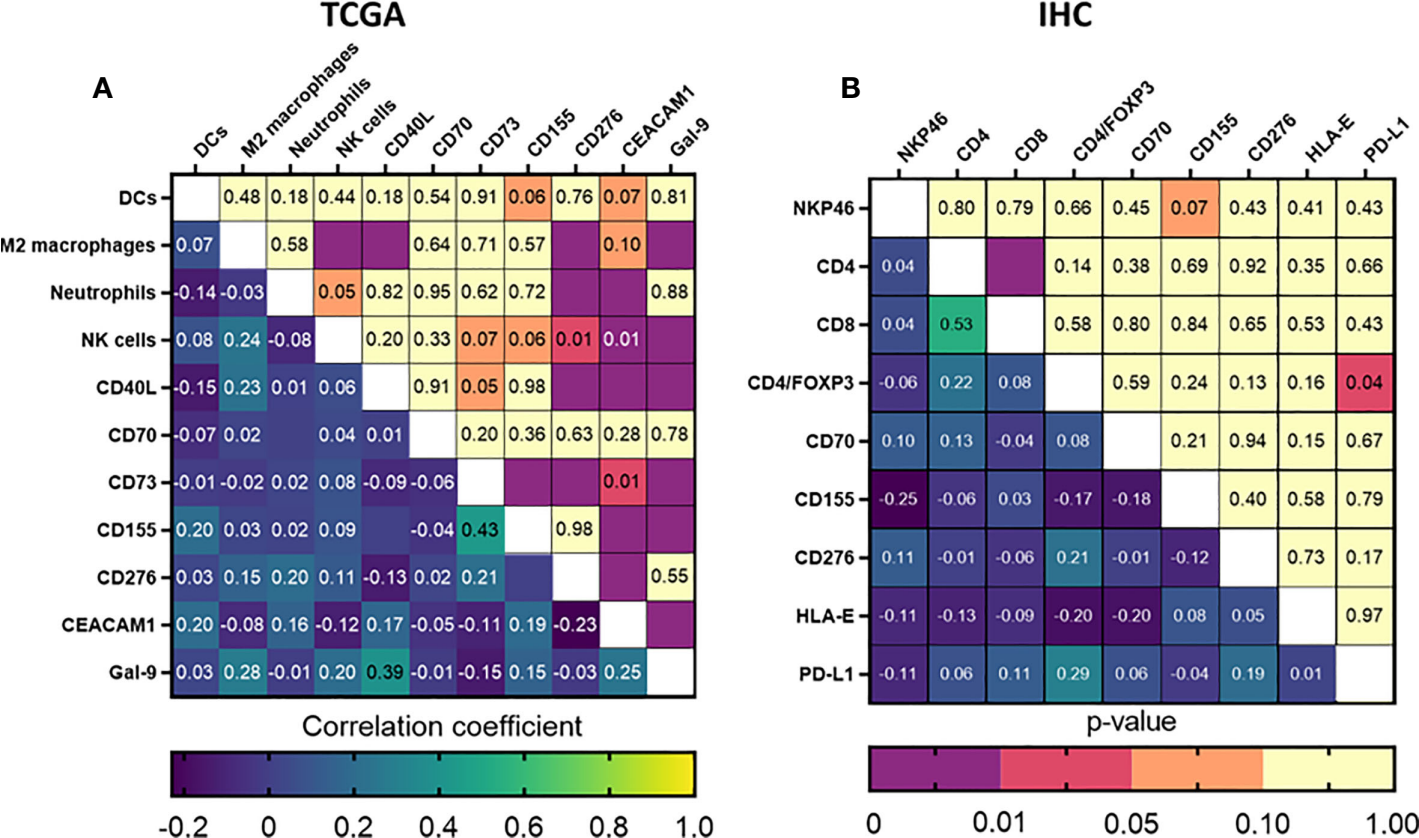
Correlation of immune composition and immune checkpoint ligands could potentially identify markers for immunotherapeutic intervention or patient stratification. Correlation matrices were generated for all prognostically relevant markers identified using our **(A)** TCGA and **(B)** UZA patient cohorts. Statistical significance was considered when p<0.05.

As a next step, we tried to further define the immune signature in HNSCC and identify patient profiles that could possibly indicate suitability for immunotherapeutic approaches. The generalized matrix showed various possible interactions (**Figure S5A**). To identify possible interactions with prognostic relevance, data of the immune composition and immune checkpoint ligand markers that were significant in the univariate analyses were combined in a correlation matrix. While TCGA showed several significant correlations, the majority of them were classified as ‘weak’ correlations, with one exception being CD155 and CD73 expression, which could be considered ‘moderately’ positive correlated (r=0.433; p<0.001) (**Figure 5A**). The IHC study cohort similarly did not result in strong correlations, but we observed a positive correlation between CD4/FoxP3 positive cells and PD-L1 expression (r=0.287; p=0.039) and a trend towards an inverse correlation between NK cell infiltration and CD155 expression (r=0.248; p=0.071) (**Figure 5B**). Based on these results, we performed multivariate cox regressions using the interaction term between these markers and only observed a significant influence of CD4/FoxP3 and PD-L1 expression on prognosis (RR: 1.47 [1.03–8.21], p=0.035), indicating a potential relevance of these markers for patient stratification. Taken together, these data suggest a possible inverse interaction between CD155 expression and NK cells infiltration in HNSCC patients and a stronger association between Treg infiltration and PD-L1 expression. Either of these interactions could be a possible immune evasion mechanism for HNSCC.

## Discussion

Immunotherapy is a rapidly developing field, with immune checkpoint inhibitors showing measurable benefit in several solid tumors (15, 16). In this regard, pembrolizumab has been approved for the treatment of recurrent and metastatic HNSCC. However, even in combination with chemotherapy, the efficacy of pembrolizumab is still quite limited, both in overall survival as well as response rates (13). The immune system is well recognized as a critical component for tumor prevention, development, and progression of cancer. Characterizing the TIME in HNSCC may therefore aid in predicting patient prognosis as well as contribute to the development of rationally designed novel cancer therapies that could outperform the current standard of care. Previously, a pan-cancer transcriptomic analysis has indicated that HNSCC is one of the highest immune infiltrated tumor types, in particular for NK cells and Tregs (36). In this study, we underlined the prognostic role of the immune composition and expression of immune checkpoint ligands in HNSCC tumors. Collectively, we have identified a negative prognostic relevance between tumor infiltration by Tregs and PD-L1 expression, and an inverse correlation between NK cell infiltration and the expression of CD155. Whether these markers can be used as prognostic indicators in a clinical setting or whether disruption of this interaction could result in a therapeutic advantage in HNSCC remains to be further explored.

In the first part of our study, the immune composition of HNSCC was characterized using two cohorts (TCGA and UZA) and two methods (RNA sequencing and IHC, respectively). Herein, our in silico data found M2 macrophages and neutrophils to be associated with a worse outcome, while DCs associated with good prognosis. Ex vivo, tumor infiltration by CD8+, NKp46+ and also CD4+/FoxP3+ immune cells correlated with a better outcome. The latter observation is somewhat in contrast with the immunosuppressive role of Tregs within the TIME.

A possible explanation may be that drivers of T cell infiltration may cause the collective infiltration of both inflammatory and immune suppressive T cells (cytotoxic, helper, memory, and regulatory T cells). Indeed, both our TCGA and IHC data showed a strong correlation between CD4+ and CD8+ T cells, both of which were correlated to Tregs in our TCGA dataset. For IHC, although not correlated, the ratio of FoxP3+ to CD8+ cells was 0.39 fold intratumorally and 0.59 fold at the tumor borders, suggesting that the prognostic effect was indeed due to the presence cytotoxic T cells. Others have reported similar findings, with a higher intratumoral Treg to CD8 ratio being indicative for a shorter OS (37). Mandal et al. showed that Tregs did not retain their independent prognostic effect following adjustment for the levels of CD8+ T cells and NK cells (36). In addition, considering our multivariate analysis suggesting a role for CD4+/FoxP3+ cells with PD-L1 expression, we postulate that this interaction might potentially play a central role towards tumor progression. In this regard, a few studies have tried to elucidate the role of PD-1/PD-L1 axis in Treg differentiation and function. While the PD-1/PD-L1 axis blocks proliferation and cytokine production and inhibits the cytotoxic activity of effector T cells, it promotes development and function of Tregs primarily through induction of the Treg-specific transcription factor FoxP3 (38, 39). Beyond their regular immunosuppressive character, Tregs may contribute to anti-PD-1/PD-L1 drug resistance through several processes and could be a one of the reasons explaining the limited clinical responses of PD-1/PD-L1 blockade (40). Therefore, the simultaneous presence of FoxP3+ Tregs together with a high dependence on PD-1/PD-L1 signaling could become a marker to stratify patient towards the applicability of PD-1/PD-L1 antagonists. On the other hand, this also encourages the simultaneous combination of anti-PD-1/PD-L1 therapies with targeting of Tregs as a possibly synergistic antitumor strategy for HNSCC tumors that are eligible for anti-PD-1/PD-L1 therapies (41, 42).

As a major source of interferon-γ during early-phase immune responses, NK cells play a critical role in tumor control as rapid-acting immune effectors (40). Pan-cancer transcriptomic analyses have revealed that HNSCC tumors are among the most highly infiltrated tumors, particularly with regards to NK cells and Tregs. Although NK cells in circulation represent about 10-15% of all immune cells (43), tumor infiltrating NK cells are present in much lower numbers. In this regard, comparable reports have presented similar findings to our observations (TCGA: 3.8%) with percentages ranging between to 1-5% (44, 45). In our UZA cohort, we identified peritumoral presence of immune cells. A clear and distinct pattern of NK cell presence at the tumor borders was noted, as observed by others as well (45). This observation could indicate that the immunosuppressive microenvironment of HNSCC might be able to both reduce the cytotoxic potential of NK cells, as observed by others (46–48), and limit the degree to which, NK cells, are able to infiltrate the tumor (49, 50). In this regard, it is known that both cytokines released by tumor cells and the presence of immunosuppressive immune cells can indeed suppress immune infiltration (10, 50, 51). As mentioned, we found an abundance of CD4+/FoxP3+ T cells in HNSCC tissues, which may coincide with the relatively low number of infiltrating NK cells. Similar findings were also reported for other tumor types (52–54). However, our IHC data indicates that patient outcome is dependent on the presence of intratumoral NK cells. This supports further development of immunotherapeutic strategies aimed at restoring NK cell functionality or improving NK cell infiltration. In the context of HNSCC, we previously reviewed how the combined use cetuximab, as an anti-EGFR ADCC-inducing antibody, with NK cell targeting immunotherapies could control tumor outgrowth and restore immune-mediated antitumor function (55).

The second part of our investigation concerned the identification of novel targets that could be of high relevance in the context of NK cell-based immunotherapies. We observed that HNSCC patients had an increased expression of a broad range of inhibitory immune checkpoint ligands in comparison to healthy tissue, which could possibly contribute to immune escape (56). Although, we also observed a significant increased expression of the apoptosis inducing TRAIL, none of these showed a significant prognostic effect, possibly as a result of increased inhibitory signaling (57). However, inhibitory receptor signaling is only capable of suppressing downstream signaling of ITAM-bearing receptors when they are clustered with ITAM-bearing receptors (58). Therefore, the lack of prognostic significance through overexpression of co-stimulatory molecules may indeed have been overpowered by the increased presence of immune checkpoint ligands on tumor cells.

Although PD-L1 and PD-L2 were differentially expressed in tumor tissue compared to healthy tissue, our UZA cohort overall showed a lower PD-L1 expression (both CPS and TPS) in comparison to the KEYNOTE trials (59, 60). In line with this observation, we did not observe a prognostic relevance for PD-L1 expression using this cohort. Whether the inclusion of a larger number of PD-L1 ‘high’ patients may have altered this observation is not clear but is something that in the future should be considered. Considering the prognostic relevance of the other immune checkpoints ligands for this cohort, CD276 (B7-H3) was one of the main immune checkpoint ligands as potentially interesting for HNSCC, identified by both our in silico and immunohistochemical observations. Originally identified as a T cell co-stimulatory molecule, CD276 (B7 family) expression on tumor cells was found to prevent T cell and NK cell activity (61) In addition, similar to our observation, high tumoral CD276 expression correlated with poor outcome as well (62). Taken together with the success that targeting other B7 family members has had, CD276-based immune checkpoint therapies are gaining attention (12, 63, 64). In this regard, inhibition of CD276 was found to reduce tumor growth, prolong survival and improve CD8+ T cell and NK cell infiltration in mouse models of colorectal cancer and osteosarcoma (65, 66). In addition, CD276 inhibition was able to reverse HNSCC cancer stem cells and metastasis. As CD276+ cells were mainly located at the peripheral tumor region (67), indicating a protective role of CD276 for immune-mediated killing, similar to our observation with NK cells. Lastly, enoblituzumab, a humanized, Fc-engineered mAb binding CD276 has recently been investigated both as single-agent as well as in combination with pembrolizumab in a phase 1 trial in HNSCC, resulting in sustained inhibition of tumor growth (68–70). Thus, further exploration of CD276-based therapeutics either as (i) ADCC-oriented mAbs (enoblituzumab), in conjunction with therapeutics that promote NK cell infiltration; or (ii) as inhibitors of the immunosuppressive NK cell signaling that could improve CD8+ T cell and NK cell infiltration, together with an ADCC-capable mAb, such as cetuximab, could achieve more effective antitumor responses.

Both of our analyses also identified CD155 as an interesting immune checkpoint ligand. CD155 (nectin-like family) has a wide variety of cellular functions, including regulation of immune cell activity through interaction with the immunomodulatory receptors DNAM-1, CD96, and TIGIT (71). Expression of CD155 on normal tissue is low, whereas many tumors have been found to overexpress CD155 (72, 73). In addition, tumor infiltrating CD8+, CD4+ T cells, and NK cells are often found to express high levels of TIGIT, the interaction of which with CD155 is known to suppress the antitumor effect of these cells. In that regard, our findings are in line with the latest *in vitro* and ex vivo reports showing a strong negative prognostic effect CD155 expression and patient survival in different tumor types (72, 74, 75). It should be noted that we are the first to report on a possible interaction between CD155 immunostaining on tumor cells and NKp46+ staining for NK cells. Based on this, we suggest that blocking the interaction between CD155 and TIGIT warrants further investigation as a new suitable treatment strategy, either as a single-agent or in combination with the current standard of care for HNSCC. Consistent with these ideas, targeting of CD155/TIGIT axis is a growing topic of interest to elicit substantial NK cell-mediated antitumor responses. In this regard, early investigations have reported increased antitumor immune responses and interferon-γ production *in vitro* and *in vivo* either through mAbs or through genetic disruption of CD155 protein expression (76, 77). As targeting of CD155/TIGIT has only recently gained great attention, further progress in the form of a better understanding of the role of CD155 in the TIME and development of new therapeutic approaches, such as combination therapies involving CD155 might support the rationale for developing therapeutic strategies targeting CD155 using neutralizing mAbs.

It is worth mentioning that our study was not without its limitations. These included the restrictions on the number of investigated markers and patients in both TCGA and UZA cohorts which could have impacted the power of our findings. Considering the technical feasibility on the number of tissue slides to be used for our analysis, we choose to employ a broad panel of possible genes in our TCGA cohort as a screening analysis and confirm their relevance on a protein level using a more defined panel. In the future, other markers could be further investigated, using preferentially a prospective patient cohort. Beyond prognosis using immune checkpoint ligands, the simultaneous investigation of immune checkpoint receptors is an important aspect to fully understand the properties of receptor-ligand interactions. Therefore, multiplex IHC, providing multidimensional co-expression analysis could further strengthen the implications for therapeutic approaches made in this study.

## Conclusions

In conclusion, our study highlights the prognostic impact of the immune composition and immune checkpoint ligand profile of HNSCC tumors. We have shown that the HNSCC TIME is well suited for various immunotherapeutic modalities that aim to restore the antitumor immunity. In this regard, CD276 and CD155 were each identified for their strong negative prognostic impact on HNSCC patients and should be further investigated in the context of checkpoint therapy. In addition, the interaction between Treg infiltration and tumor PD-L1 expression suggest that improved inhibition of this immunosuppressive cell type may achieve greater clinical activity. All together, we suggest that, in order to enhance antitumor responses, current HNSCC research should focus on the modulation of the TIME to support infiltration of tumor-associated NK cells and CD8+ T cells. This may be achieved through implementation of combined targeting different immunotherapies using prognostically relevant immune checkpoint ligands or through combination with other therapeutic modalities.

## Data availability statement

The datasets generated during and/or analysed during the current study are not publicly available due to agreements with ethical commitee but can be made available from the corresponding author on reasonable request and agreements from the involved ethical commitees.

## Ethics statement

The studies involving human participants were reviewed and approved by Ethics Committee of the Antwerp University Hospital (BB20014) and the Ethics Committee of the University of Antwerp (14/47/480). The patients/participants provided their written informed consent to participate in this study.

## Author contributions

HB, IDP, JJ, JBV, JDW and AW designed and conceptualized the presented idea. MP, PP, JBV provided the clinical framework for data acquisition. HB, EF, JDW and AW designed the computational framework. HB, VS, CH, HWL, and HL carried out experiments and acquisition of patient data. EF verified the analytical methods. IDP, JJ, JDW and AW supervised the study HB, JDW and AW wrote the original draft. All authors reviewed, edited and approved the manuscript.

## Funding

The research was funded by Kom op tegen Kanker (Stand up to Cancer), the Flemish cancer society (OZ7886). Part of this research was funded by donations from different donors, including Dedert Schilde vzw and Mr Willy Floren.

## Acknowledgments

The human biological material used in this publication was provided by the UZA Tumor bank, Antwerp University Hospital, Belgium, which is funded by the National Cancer Plan. The protocol for analyses was approved by the Ethics Committee of the Antwerp University Hospital (reference number: BB20014)/University of Antwerp (reference number: 14/47/480) in accordance with the latest version of the WMA Declaration of Helsinki.

## Conflict of interest

The authors declare that the research was conducted in the absence of any commercial or financial relationships that could be construed as a potential conflict of interest.

## Publisher’s note

All claims expressed in this article are solely those of the authors and do not necessarily represent those of their affiliated organizations, or those of the publisher, the editors and the reviewers. Any product that may be evaluated in this article, or claim that may be made by its manufacturer, is not guaranteed or endorsed by the publisher.
