# Supplementary material for: The prognostic impact of the immune signature in head and neck squamous cell carcinoma
Source: Front Immunol. 2022 Oct 4;13:1001161. doi: 10.3389/fimmu.2022.1001161 (PMC9576890; doi:10.3389/fimmu.2022.1001161)
Supplement: Supplementary file 1 [file DataSheet_1.docx]

# Supplementary Material & Legends

**Supplementary Table S1** Forest plot of the risk ratios (RR) that different patient characteristics within the TCGA and UZA cohorts have.

| Patient Characteristics | OS RR (95% CI) | |  | p-value |
| --- | --- | --- | --- | --- |
|  | TCGA (●) | IHC (■) |  |  |
| Gender |  |  |  |  |
| Female |  | 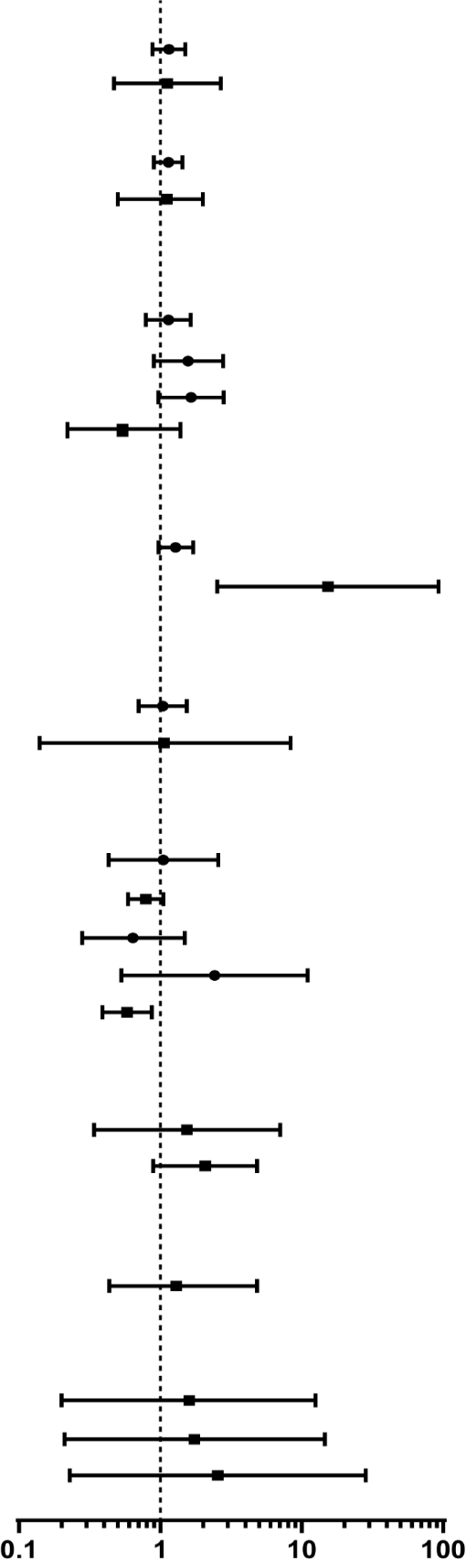 |  |  |
| Male | 1.15 (0.88 – 1.50) | 1.12 (0.47 – 2.68) |  | (●) 0.312 |
|  |  |  |  | (■) 0.797 |
| Age |  |  |  |  |
| Median | 1.14 (0.90 – 1.43) | 1.11 (0.50 – 2.00) |  | (●) 0.276 |
|  |  |  |  | (■) 0.784 |
| Stage |  |  |  |  |
| I |  |  |  |  |
| II | 1.14 (0.79 – 1.64) | - |  | (●) 0.474 |
| III | 1.57 (0.90 – 2.78) | - |  | (●) 0.113 |
| IV | 1.65 (0.97 – 2.80) | 0.54 (0.22 – 1.39) |  | (●) 0.065 |
|  |  |  |  | (■) 0.205 |
| Smoking |  |  |  |  |
| No |  |  |  |  |
| Yes | 1.28 (0.97 – 1.71) | 15.33 (2.53 – 92.75) |  | (●) 0.253 |
|  |  |  |  | (■) 0.003 |
| Alcohol |  |  |  |  |
| No |  |  |  |  |
| Yes | 1.04 (0.70 – 1.54) | 1.06 (0.14 – 8.32) |  | (●) 0.840 |
|  |  |  |  | (■) 0.956 |
| Primary site |  |  |  |  |
| Larynx |  |  |  |  |
| Oral cavity | 1.05 (0.43 – 2.57) | 0.79 (0.59 – 1.05) |  | (●) 0.912 |
|  |  |  |  | (■) 0.105 |
| Hypopahrynx | 0.64 (0.28 – 1.49) | - |  | (●) 0.305 |
| Oropharynx | 2.42 (0.53 – 11.04) | 0.58 (0.39 – 0.87) |  | (●) 0.251 |
|  |  |  |  | (■) 0.008 |
| Time |  |  |  |  |
| Hot |  |  |  |  |
| Excluded | - | 1.54 (0.34 – 7.03) |  | (■) 0.574 |
| Deserted | - | 2.08 (0.89 – 4.83) |  | (■) 0.089 |
| Tissue Type |  |  |  |  |
| Primary |  |  |  |  |
| Relapse | - | 1.30 (0.44 – 3.85) |  | (■) 0.639 |
| Differentiation |  |  |  |  |
| Well |  |  |  |  |
| Moderate | - | 1.60 (0.20 – 12.5) |  | (■) 0.654 |
| Poor | - | 1.74 (0.21 – 14.57) |  | (■) 0.609 |
| Undifferentiated | - | 2.55 (0.23 – 28.37) |  | (■) 0.446 |
|  |  |  |  |  |
|  |  |  | 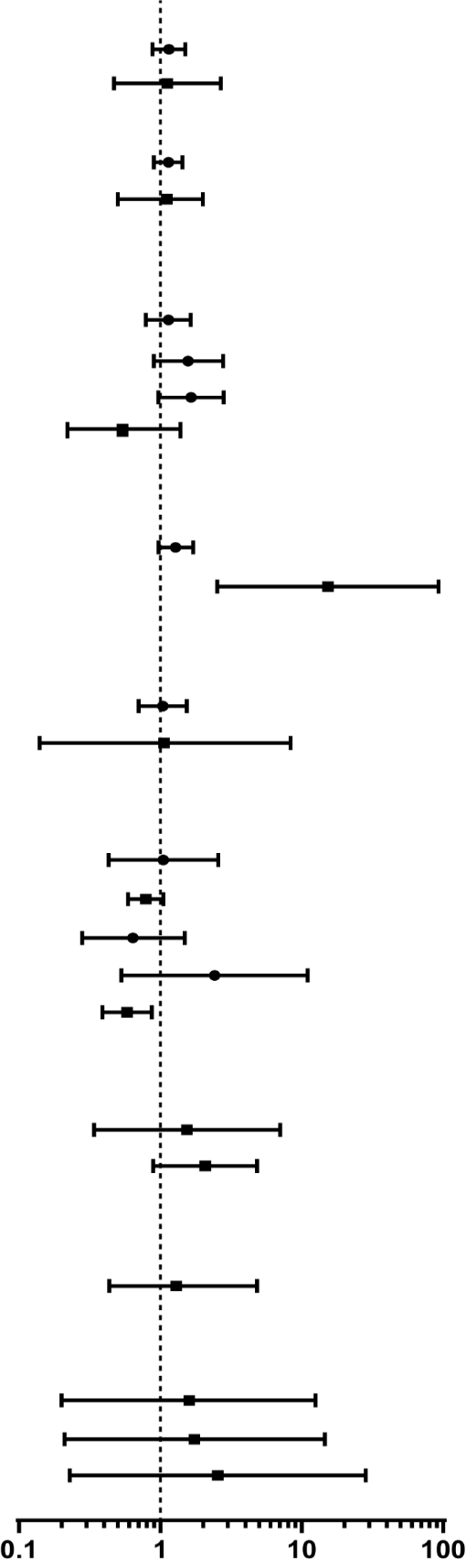 |  |
| CI; Confidence interval, OS; Overall survival, RR; Risk Ratio | | | | |

**Supplementary Table S2** Immune composition and DEGs with prognostic relevance in TCGA cohort.

|  | **OS RR** | **95% CI** | ***p-value*** |
| --- | --- | --- | --- |
| Immune composition |  |  |  |
| M2 macrophages | 1.39 | 1.05 – 1.86 | 0.023 |
| Neutrophils | 1.35 | 1.02 – 1.79 | 0.031 |
| DC | 0.41 | 0.21 – 0.81 | 0.010 |
| Immune checkpoint ligands |  |  |  |
| CD155 | 1.59 | 1.15 – 2.20 | 0.005 |
| CD73 | 1.55 | 1.18 – 2.02 | 0.002 |
| EGFR | 1.54 | 1.11 – 2.15 | 0.011 |
| CD276 | 1.44 | 1.10 – 1.89 | 0.007 |
| CD70 | 1.43 | 1.04 – 1.97 | 0.028 |
| VSIG-3 | 1.43 | 1.08 – 1.88 | 0.012 |
| Axl | 1.39 | 1.06 – 1.83 | 0.017 |
| CEACAM1 | 0.69 | 0.53 – 0.91 | 0.008 |
| CI; Confidence interval, DC; Dendritic cell, OS; Overall survival, RR; Risk ratio  Legend: Multivariate cox-proportional hazards were used to estimate risk ratios, accounting for ‘primary tumor site‘ in univariate association (p<0.05). | | | |

Supplementary Table S3 Genes involved in immune checkpoint regulation which are differentially expressed (DEGs) in HNSCC tumor samples compared to normal tissue.

| Gene | Log²FC (T/N) | *p-value* |
| --- | --- | --- |
| OX40L | 3.361 | ˂0.001 |
| CD70 | 3.328 | ˂0.001 |
| ULBP1 | 3.041 | ˂0.001 |
| RANKL | 2.549 | ˂0.001 |
| IDO1 | 2.471 | ˂0.001 |
| CD80 | 2.394 | ˂0.001 |
| PD-L2 | 2.325 | ˂0.001 |
| MICB | 2.296 | ˂0.001 |
| CD276 | 2.236 | ˂0.001 |
| CD73 | 2.017 | ˂0.001 |
| ULBP2 | 1.961 | ˂0.001 |
| GITR-L | 1.835 | ˂0.001 |
| VSIG3 | 1.617 | ˂0.001 |
| CD30 | 1.603 | ˂0.001 |
| PD-L1 | 1.534 | ˂0.001 |
| EGFR | 1.510 | ˂0.001 |
| HLA-C | 1.439 | ˂0.001 |
| TRAIL | 1.414 | ˂0.001 |
| ULBP3 | 1.386 | ˂0.001 |
| IL-15RA | 1.363 | ˂0.001 |
| Axl | 1.327 | ˂0.001 |
| CD86 | 1.280 | ˂0.001 |
| CD155 | 1.237 | ˂0.001 |
| MICA | 1.139 | ˂0.001 |
| CD137L | 1.100 | ˂0.001 |
| TYRO3 | -0.655 | 0.002 |
| ULBP4 | -1.627 | ˂0.001 |
| CEACAM1 | -2.268 | ˂0.001 |
| Abbreviations: FC; Fold change, T; Tumor, N; Normal | | |

**Supplementary Table S4** Prognostic relevance of the immune composition and immune checkpoint ligand expression of the UZA cohort.

|  | **Location** | **OS RR** | **95% CI** | ***p-value*** |
| --- | --- | --- | --- | --- |
| Immune composition |  |  |  |  |
| OS |  |  |  |  |
| CD4 | Tumor | 3.98 | 1.34 – 11.82 | 0.013 |
| CD8 | Tumor | 0.31 | 0.011 – 0.97 | 0.044 |
| CD4/FoxP3 | Tumor | 0.20 | 0.04 – 0.55 | 0.016 |
| CD4/FoxP3 | Stroma | 3.05 | 1.03 – 9.06 | 0.032 |
| NKp46 | Tumor | 0.55 | 0.26 – 0.84 | 0.037 |
| PFS |  |  |  |  |
| CD4 | Tumor | 2.31 | 1.09 – 4.93 | 0.012 |
| CD4/FoxP3 | Tumor | 0.26 | 0.13 – 0.55 | 0.005 |
| Immune checkpoint ligands |  |  |  |  |
| OS |  |  |  |  |
| CD70 |  | 6.94 | 1.21 – 39.68 | 0.029 |
| CD276 |  | 4.31 | 1.03 – 23.25 | 0.031 |
| CD155 |  | 4.27 | 1.14 – 16.08 | 0.0322 |
| PFS |  |  |  |  |
| CD276 |  | 4.92 | 1.03 – 23.25 | 0.031 |
| PD-L1 |  | 3.69 | 1.31 – 10.35 | 0.009 |
| CI; Confidence interval, OS; overall survival. PFS; Progression free survival  Legend: Multivariate cox-proportional hazards were used to estimate risk ratios, accounting for ‘primary tumor site‘, ‘age’ and ‘smoking’ in univariate association (p<0.05) | | | | |

Supplementary Figure S1 Clinicopathological parameters associate with immune composition of a TCGA dataset. The associations between immune composition and various clinicopathological parameters were univariately analyzed using T-test (level=2) or One way ANOVA (level>2). Statistical significance is reported for the following levels: *p<0.05, **p<0.01, ***p<0.001.

Supplementary Figure S2 Clinicopathological parameters associate with immune expression of a TCGA dataset. The associations between immune checkpoint ligand expression and various clinicopathological parameters were univariately analyzed using T-test (level=2) or One way ANOVA (level>2). Statistical significance is reported for the following levels: *p<0.05, **p<0.01, ***p<0.001.

Supplementary Figure S3 Clinicopathological parameters associate with immune composition of the UZA cohort. The associations between immune composition and various clinicopathological parameters were univariately analyzed using Mann-Whitney U (level=2) or Kruskal-Wallis (level>2). Statistical significance is reported for the following levels: *p<0.05, **p<0.01, ***p<0.001.

Supplementary Figure S4 Clinicopathological parameters associate with immune expression of the UZA cohort. The associations between immune checkpoint ligand expression and various clinicopathological parameters were univariately analyzed using Mann-Whitney U (level=2) or Kruskal-Wallis (level>2). Statistical significance is reported for the following levels: *p<0.05, **p<0.01, ***p<0.001.

**Supplementary Figure S5** Correlation of immune composition and immune checkpoint ligand expression using TCGA and UZA patient cohorts. Correlation matrices were generated between all immunomarkers selected for analysis in our study from both **(A)** our TCGA **(B)** as well as our UZA patient cohorts. Statistical significance was considered when p<0.05.
