# Supplementary figures and images for: The prognostic impact of the immune signature in head and neck squamous cell carcinoma
Source: Front Immunol. 2022 Oct 4;13:1001161. doi: 10.3389/fimmu.2022.1001161 (PMC9576890; doi:10.3389/fimmu.2022.1001161)

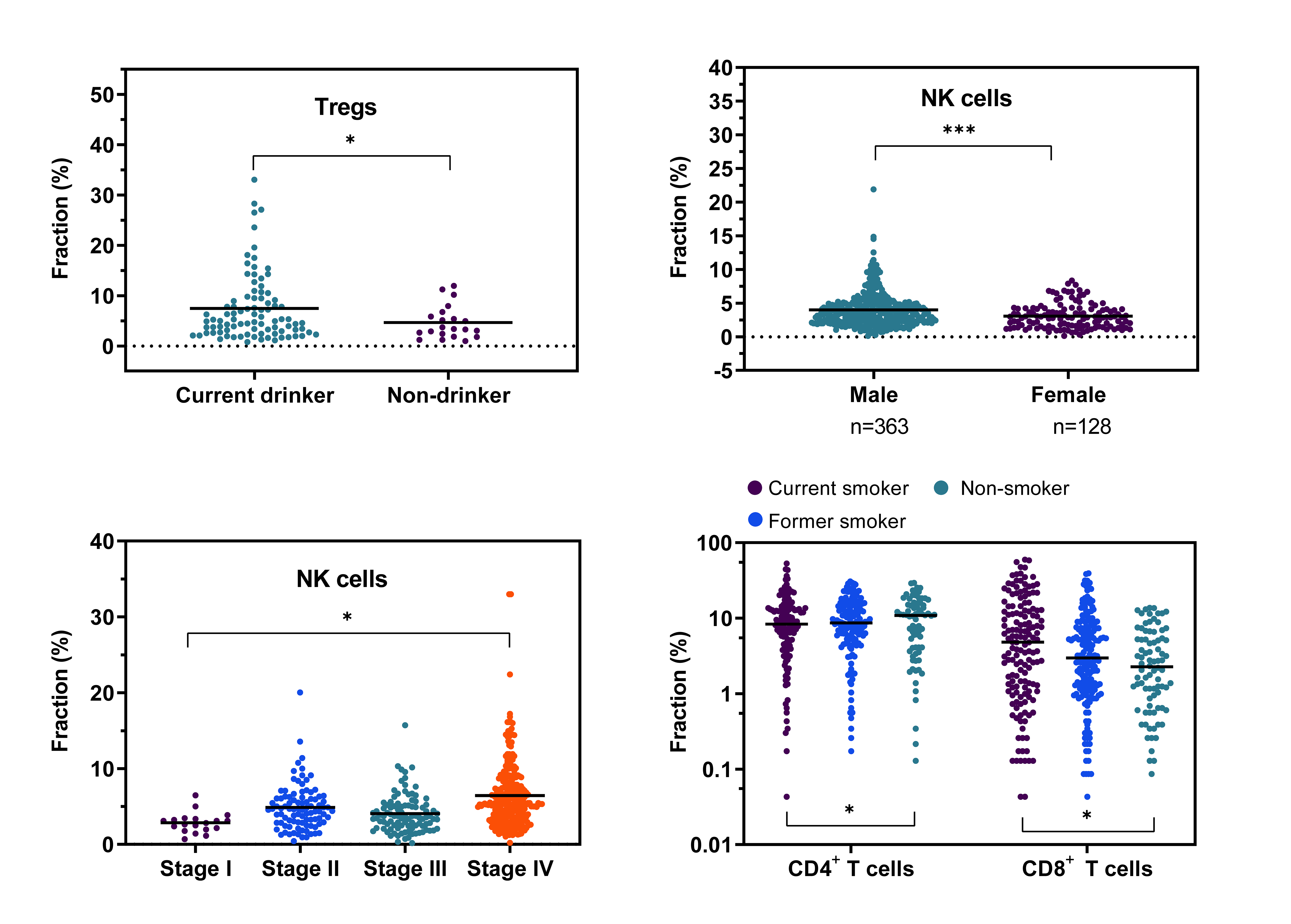

Supplement: Supplementary file 2 [file Image_1.tif]

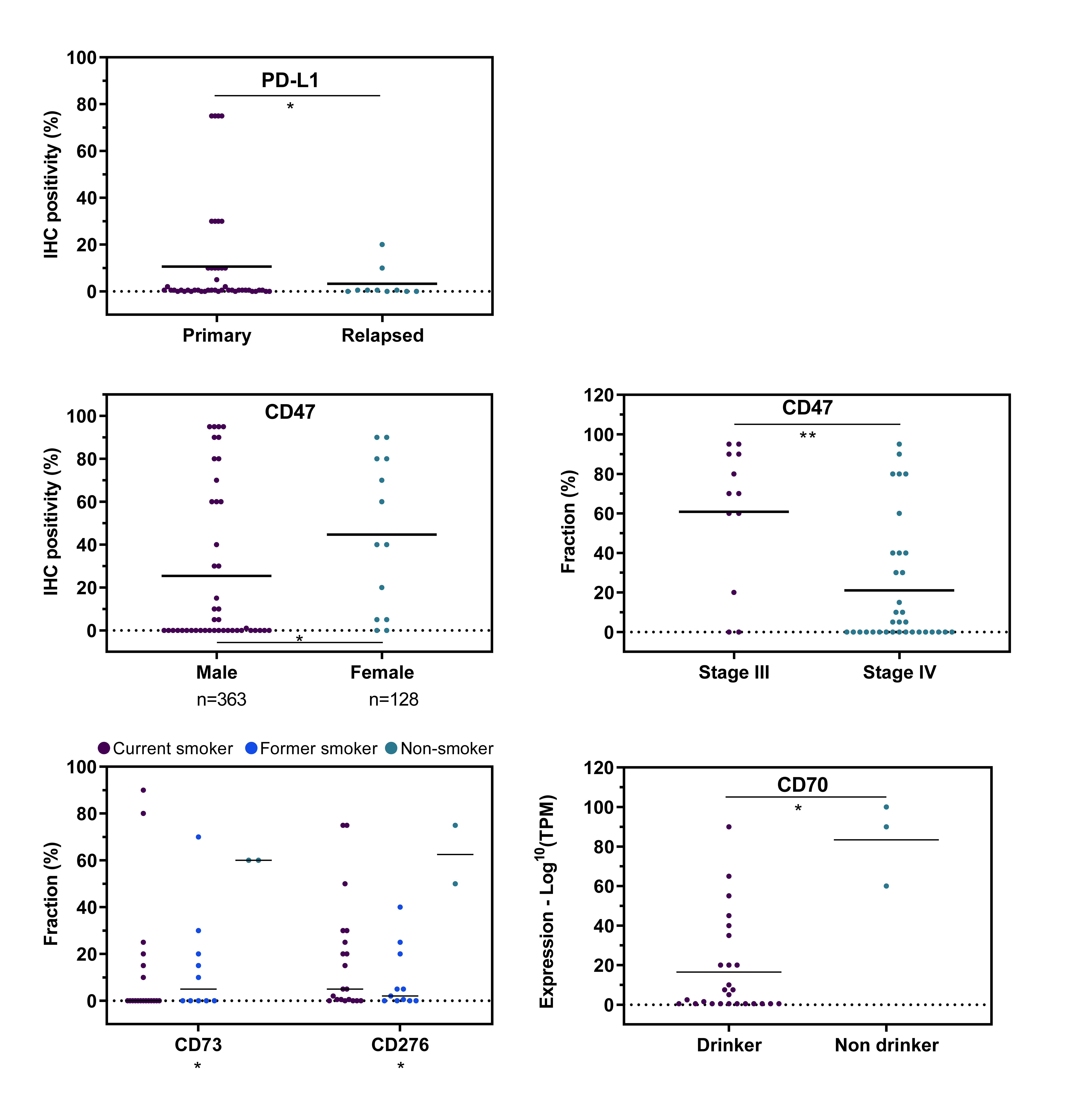

Supplement: Supplementary file 5 [file Image_4.tif]

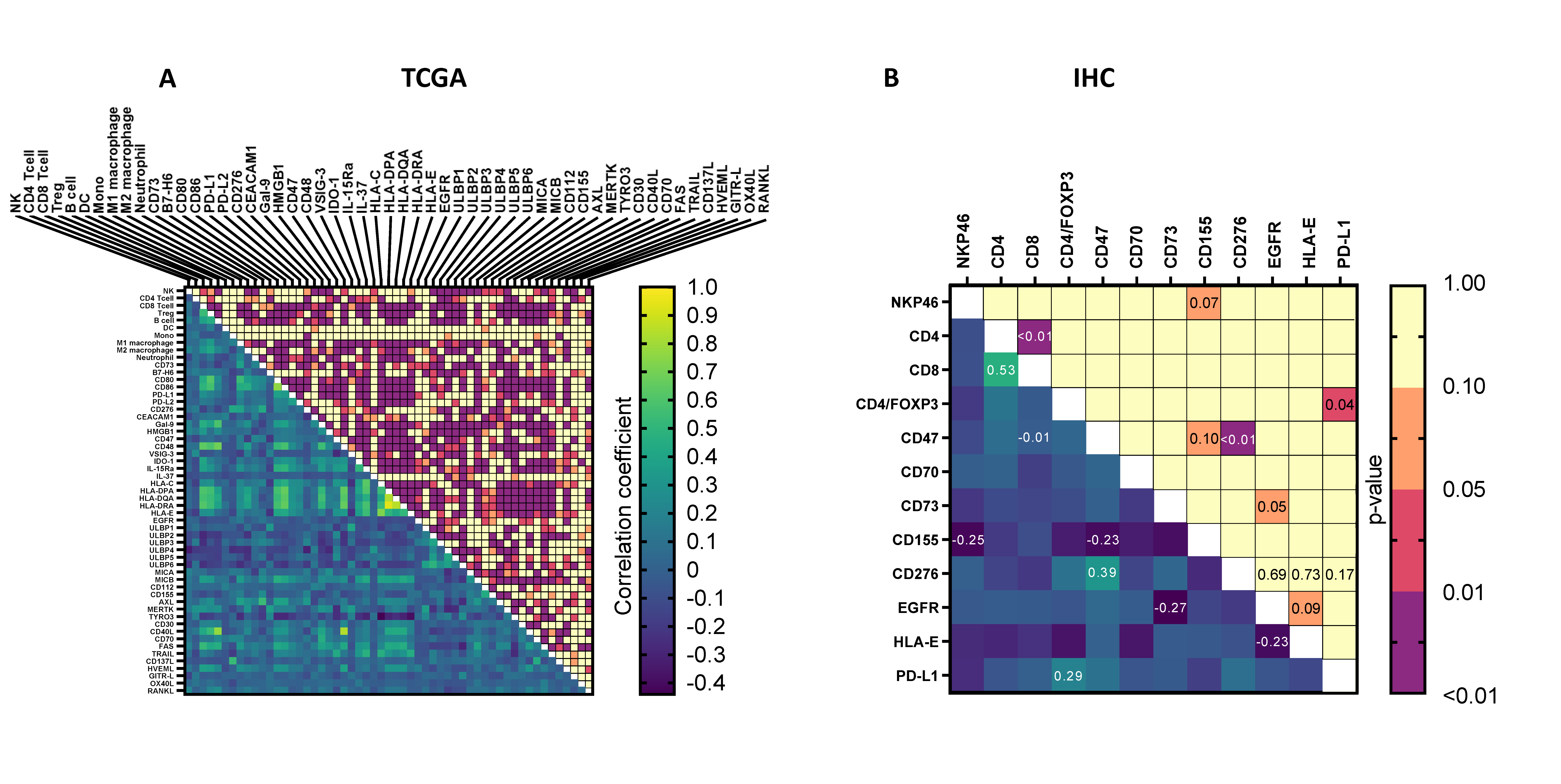

Supplement: Supplementary file 6 [file Image_5.tif]
